# A genome-wide association study explores the genetic determinism of host resistance to S*almonella pullorum* infection in chickens

**DOI:** 10.1186/s12711-019-0492-4

**Published:** 2019-09-18

**Authors:** Xinghua Li, Changsheng Nie, Yuchen Liu, Yu Chen, Xueze Lv, Liang Wang, Jianwei Zhang, Kaiyang Li, Yaxiong Jia, Liping Ban, Zhonghua Ning, Lujiang Qu

**Affiliations:** 1 State Key Laboratory of Animal Nutrition, Department of Animal Genetics and Breeding, National Engineering Laboratory for Animal Breeding, College of Animal Science and Technology, China Agricultural University, Beijing, China; 2 Beijing Municipal General Station of Animal Science, Beijing, China; 3 Institute of Animal Sciences, Chinese Academy of Agricultural Sciences, Beijing, China; 4 Department of Grassland Science, China Agricultural University, Beijing, China

## Abstract

**Background:**

*Salmonella* infection is a serious concern in poultry farming because of its impact on both economic loss and human health. Chicks aged 20 days or less are extremely vulnerable to *Salmonella pullorum* (SP), which causes high mortality. Furthermore, an outbreak of SP infection can result in a considerable number of carriers that become potential transmitters, thus, threatening fellow chickens and offspring. In this study, we conducted a genome-wide association study (GWAS) to detect potential genomic loci and candidate genes associated with two disease-related traits: death and carrier state.

**Methods:**

In total, 818 birds were phenotyped for death and carrier state traits through a SP challenge experiment, and genotyped by using a 600 K high-density single nucleotide polymorphism (SNP) array. A GWAS using a single-marker linear mixed model was performed with the GEMMA software. RNA-sequencing on spleen samples was carried out for further identification of candidate genes.

**Results:**

We detected a region that was located between 33.48 and 34.03 Mb on chicken chromosome 4 and was significantly associated with death, with the most significant SNP (rs314483802) accounting for 11.73% of the phenotypic variation. Two candidate genes, *FBXW7* and *LRBA*, were identified as the most promising genes involved in resistance to SP. The expression levels of *FBXW7* and *LRBA* were significantly downregulated after SP infection, which suggests that they may have a role in controlling SP infections. Two other significant loci and related genes (*TRAF3* and *gga*-*mir*-*489*) were associated with carrier state, which indicates a different polygenic determinism compared with that of death. In addition, genomic inbreeding coefficients showed no correlation with resistance to SP within each breed in our study.

**Conclusions:**

The results of this GWAS with a carefully organized *Salmonella* challenge experiment represent an important milestone in understanding the genetics of infectious disease resistance, offer a theoretical basis for breeding SP-resistant chicken lines using marker-assisted selection, and provide new information for salmonellosis research in humans and other animals.

## Background

*Salmonella* infection is a serious concern in poultry farming. On the one hand, systemic salmonellosis results in considerable animal mortality and reduced poultry production. On the other hand, poultry is a major global reservoir of nontyphoidal *Salmonellae*, which is one of the most important pathogens that cause foodborne illnesses [1]. Pullorum disease is an acute poultry infectious disease that is caused by the chicken-restricted facultative intracellular gram-negative bacterium, *Salmonella pullorum* (SP). This disease usually results in high mortality of chicks less than 20 days old, especially in developing countries where cleaning and disinfection procedures are usually not effective [2]. SP infection generally leads to three disease outcomes: the most susceptible birds die within about 2 to 20 days showing typical SP infection symptoms such as hepatosplenomegaly, white diarrhea and cecal cores [3]; some chicks survive by clearing the pathogen through a series of immune responses; and other chicks develop a carrier state with SP present in their splenic macrophages for a long period of time [4]. These carriers can transmit the pathogen to other chickens horizontally or to their offspring via the eggs [5].

In many developed countries, the pullorum disease has been eradicated from commercial flocks by culling infected birds. However, this method does not work well in most developing countries due to the emergence of novel bacterial strains [6], poor hygienic conditions, and limited technology; in addition, there are restrictions on the use of antibiotics in food animal production. Thus, there is need for an alternative sustainable strategy to control the disease in farm animals. In addition to novel vaccines and food additives, selective breeding of animals based on the development of chicken genomic data is becoming a promising approach to improve their resistance to infectious diseases [7].

Host genetic factors have been reported to play an important role in the resistance of animals to *Salmonella* infection in many studies [8–14]. Previously, we estimated the heritability of the death and carrier state traits based on an elaborately designed challenge experiment [3]. The results showed low-to-moderate heritabilities (0.09 to 0.32) in different chicken lines, which means these traits are heritable. However, the molecular mechanism that underlies the genetic resistance to SP remains largely unknown. In recent years, genome-wide association studies (GWAS) have been widely used to identify the genetic architecture of many disease traits in chickens [15–18]. However, only a few GWAS have been carried out on infectious diseases because it is difficult and expensive to obtain accurate phenotypes for large populations; furthermore, the results of an infection are affected by many factors such as bacterial dosage, maternal antibodies, and the environment [19], which are difficult to control.

To the best of our knowledge, no large-scale GWAS has been performed to identify genomic loci and candidate genes for death and carrier state, through a carefully organized SP challenge test. In this study, 818 pure-bred chicks were genotyped with a commercial 600 K high-density single nucleotide polymorphism (SNP) array [20]. A GWAS using a single-marker linear mixed model and 302,927 SNPs allowed us to detect genomic regions that are associated with resistance to SP. The identified candidate genes were evaluated based on their functional annotation and expression level. The potential mechanisms of these genes in immunity to infection in chickens are discussed.

## Methods

### Animals and phenotyping

For this study, 842 chicks from three pure lines were available, namely 384 Rhode Island Red, 381 Dwarf Chicken, and 77 Beijing You individuals. Rhode Island Red (RIR) is an intensively selected commercial breed, Dwarf Chicken (DW) is a synthetic layer line, and Beijing You (BY) is a Chinese local chicken breed. These animals all came from our previous SP challenge experiment [3]. Briefly, SP pathogen-free and antibody-free chicks were orally inoculated with 4.8 × 10^7^ colony forming units (CFU) SP strain 533 culture at 4 days of age and then raised in negative pressure isolators up to 40 days of age. All chicks had free access to sterile water and food, and the environment was managed such that it remained the same for all the birds in the experiment. Three different disease-related traits, i.e. death, clearance and carrier-state, were measured and used as SP resistance phenotypes. First, chicks that died with visible signs of SP infection symptoms (white diarrhea and splenomegaly) were classified as the most susceptible. In the case of chicks that survived the challenge, the carrier-state was analyzed by plating spleen homogenates on MacConkey agar medium under sterile conditions to determine bacterial load. The identity of the pathogen was confirmed by Sanger sequencing of the SP specific *ipaJ* gene [21] after PCR amplification with the following primers: sense 5′-ATTAACAGGAGGAGGCTGG-3′; antisense 5′-CCATTCCCAAAAGCCTGCAT-3′. More details on how the population was established and on the bacterial challenge process are in our previous report [3].

### Genotyping, quality control and imputation

We isolated individual genomic DNA from blood or muscle samples by the classical phenol–chloroform procedure. DNA integrity was verified by agarose gel electrophoresis and purity was checked by A260/280 ratio using a NanoDrop 2000 spectrophotometer (Thermo Fisher Scientific™). In total, 842 qualified individual genomic DNA samples were genotyped using the Affymetrix 600 K chicken high-density array (Affymetrix, Inc. Santa Clara, CA, USA). For SNP calling and initial quality control, the raw genotyping data (CEL files) were analyzed by using the software Axiom Analysis Suite 3.1 following the Best Practices Workflow. Only the samples with a dish quality control (DQC) of 0.82 or more and a call rate higher than 95% were retained for subsequent analyses. The SNP QC metrics were set to the default values recommended by Affymetrix, except that only “PolyHighResolution” SNPs were included in our analysis. Furthermore, we excluded SNPs with unknown or repeated physical positions with an ad hoc R script. SNPs on the sex chromosomes were also discarded because the current statistical methods are not powerful enough to detect associations between phenotypes and sex-related genotypes. After these QC steps, 818 samples and 452,291 SNPs remained. A Hardy–Weinberg equilibrium (HWE) test was conducted within each breed, and 62,450 variants that deviated from HWE (P < 1 × 10^−5^) were filtered out. To increase the power of the association analysis, we removed 85,140 SNPs because of their low level of variation among subpopulations (i.e. SNPs that were monomorphic in any one of the three breeds), and 1774 SNPs with a minor allele frequency (MAF) lower than 0.05 using PLINK v1.90 [22]. Missing genotypes were imputed based on information from the remaining SNP genotypes for each subpopulation separately, according to the software Beagle Version 4 [23]. In total, 818 samples and 302,927 SNPs were included in the subsequent genome-wide association analysis.

### Statistical analysis

Population structure and relatedness are major sources of confounding effects in genetic association studies [24]. The most popular method for GWAS that include related individuals is the linear mixed model (LMM) method because of its effectiveness in controlling population stratification bias and reducing the inflation from many small genetic effects (polygenic background) [25–31]. In this study, we assessed population structure by conducting a principal component analysis (PCA) implemented in the PLINK package. Considering that clusters of SNPs in high linkage disequilibrium may bias the PCA results, first we pruned the full SNP set to 23,870 independent SNPs using the—indep-pairwise 25 5 0.2 command parameters in PLINK. Then, we used these unlinked SNPs to calculate the top three principal components (PC) that were used as covariates in the mixed model. Furthermore, a pairwise kinship matrix was built using the pruned SNPs.

A single-marker univariate linear mixed model was used for testing associations between the results of SP infection and the qualified SNPs. The disease phenotype was divided into two binary traits: death (200 deaths vs. 618 survivals) and carrier-state (161 carriers vs. 457 clearance). Both death and carrier-state were analyzed using the following model:

$$ {\mathbf{y}} = {\mathbf{W}}{\varvec{\upalpha}} + {\mathbf{x}}{\varvec{\upbeta}} + {\mathbf{u}} + {\varvec{\upvarepsilon}}, $$

where ${\mathbf{y}}$ denotes the trait values for death or carrier-state, namely either ‘0’ or ‘1’; ${\mathbf{W}}$ is a matrix of covariates (i.e. fixed effects that contain the top three PC, genotyping batch and a column of 1s) that control population structure and batch effect; ${\varvec{\upalpha}}$ is a vector of corresponding coefficients that includes the intercept; ${\mathbf{x}}$ is a vector of SNP genotypes; ${\varvec{\upbeta}}$ is the corresponding effect of SNPs; ${\mathbf{u}}$ is a vector of random polygenic effects with a covariance structure that follows a normal distribution ${\mathbf{u}}\sim {\text{N}}\left( {0,{\mathbf{K}}{\text{V}}_{\text{g}} } \right)$, where ${\mathbf{K}}$ is a genomic relationship matrix derived from independent SNPs and ${\text{V}}_{\text{g}}$ is the polygenic additive variance; and ${\varvec{\upvarepsilon}}$ is a vector of random errors. The association analysis was performed using the GEMMA v0.96 software [30]. The Wald test statistic ${\text{F}}_{\text{wald}} = \hat{\beta }^{2} /Var\left( {\hat{\beta }} \right)$ was used to test the null hypothesis $\beta = 0$ for each SNP. The Manhattan and quantile–quantile (Q–Q) plots were drawn with the “qqman” package in R. Moreover, correction for population stratification was evaluated by calculating the genomic inflation factor λ with the “GenABEL” package [32].

We calculated genome-wide significance P-value thresholds with the simpleM method implemented in a R script for multiple testing correction [33]. simpleM calculation resulted in 72,648 independent effective tests and the genome-wide and suggestive significance values were then calculated as 6.88 × 10^−7^ (0.05/72,648) and 1.38 × 10^−5^ (1.00/72,648), respectively. The contribution of significant SNPs to the phenotypic variance was estimated by a restricted maximum likelihood (REML) method implemented in the GCTA v1.91 software [34]. Linkage disequilibrium (LD) analysis was performed for the significant SNPs using the solid spin algorithm implemented in Haploview version 4.2 [35].

### Identification of candidate genes

Candidate genes for resistance to SP were identified based on functional annotation and expression level data. First, we performed functional annotation by searching for candidate genes within 500 kb regions on either side of the lead significant SNP with the help of the Variant Effect Predictor [36] and Biomart tools supported by Ensembl (http://www.ensembl.org) using the Gallus_gallus-5.0 genes dataset. Then, we investigated the biological functions of these candidate genes on PubMed (https://www.ncbi.nlm.nih.gov/pubmed).

### Gene expression analysis

The gene expression levels in the spleen of infected and mock-infected birds (54 samples) were determined by RNA-Seq (three replicates) at three time points [4, 10 and 21 days post-infection (dpi)] for the three breeds. Spleen tissue was sampled and immediately preserved in RNAlater (Ambion) at room temperature for 24 h and then at − 20 °C until RNA extraction. Total RNA was extracted using the Trizol reagent (Invitrogen, Carlsbad, CA, USA) following the manufacturer’s protocol. Libraries for each sample were prepared and sequenced on an Illumina HiSeq 2500 platform (Illumina Inc., San Diego, CA, USA), and 150 bp paired-end reads were generated. Raw reads were filtered and trimmed using fastp (version 0.19.1) [37]. Clean reads were mapped to the chicken reference genome (galGal5) using the HISAT2 program [38]. Reads mapped to a gene were counted with featureCounts [39] against the gene annotation from Ensembl database. The gene counts were normalized by the Bioconductor package DESeq2 [40] and differential expression P-values of candidate genes were then calculated with default parameters. To evaluate the correlation between time and gene expression levels of the control group, we conducted regression analysis by fitting the linear model with the function implemented in the R software version 3.5.1 (Foundation for Statistical Computing, Vienna, Austria).

### Genomic inbreeding analysis

Inbreeding has long been reported to affect fitness traits such as resistance to disease [41–45]. To investigate the relationship between inbreeding and resistance to SP, we estimated the genomic inbreeding coefficients by measuring the genome-wide distribution of runs of homozygosity (ROH) using the PLINK software. The genomic inbreeding coefficient F_ROH_ was calculated as the proportion of the genome which is found in runs of homozygosity. The total physical length of all autosomes from the first to the last SNP is 930,016,100 bp. A ROH is defined as a segment of consecutive homozygous DNA that meets the following criteria: (1) a minimum size of 1000 kb and at least 50 homozygous SNPs; (2) a maximum of five missing SNPs allowed in a ROH; (3) a maximum of one heterozygous SNP per ROH, so that ROH segments are not disrupted by an occasional heterozygous SNP; and (4) a maximum gap between SNPs of 1000 kb to ensure that SNP density does not affect a ROH.

## Results

### Phenotype statistics and population structure

**Fig. 1:**
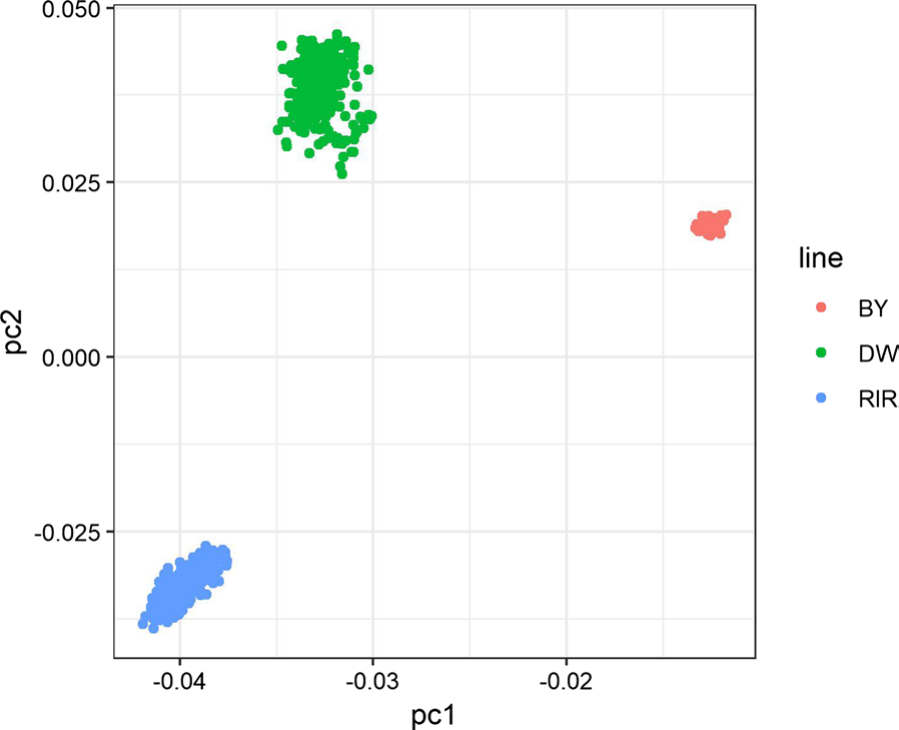
PCA plot for population structure. The red, green and blue dots represent BY, DW and RIR individuals, respectively

The details of the results of the SP challenge were previously reported [3]. Briefly, mortality rates for the RIR, BY, and DW chicks reached 25.1, 8.3, and 22.7%, respectively, and the corresponding carrier-state levels in the spleens were equal to 17.9, 0.6, and 15.8%, which indicate that BY chicks are more resistant to SP infection than DW and RIR chicks. Only part of the chicks that participated in the SP challenge test were genotyped. Since nearly all the BY chicks that survived could clear the pathogen (except three), we did not genotype the carriers in this breed. In the group of chicks that died, only the individuals that had conclusive symptoms and for which high quality DNA was available were genotyped. Furthermore, in the group of chicks that cleared the pathogen, we removed randomly some individuals to match the corresponding phenotype proportion. In total, 842 samples were genotyped and after a series of strict quality control procedures, 24 samples were eliminated because of a low genotyping call rate. Thus, the final GWAS population consisted of 818 samples (392 males and 426 females). The phenotypic composition of each line is given in Table 1. Based on the PCA plot (Fig. 1), three subpopulations are clearly distinguished, which indicates that population stratification can be accounted for in the linear mixed model of the GWAS by including these principal components as covariates in the analysis.

**Table 1:** Phenotype composition of the three chicken lines used in the GWAS

Breed	Died	Carrier state	Clearance	Total
BY	27	0	50	77
DW	89	77	198	364
RIR	84	84	209	377
Total	200	161	457	818

*BY* Beijing You, *DW* Dwarf Chicken, *RIR* Rhode Island Red. These are the number of chicks that died, carried and cleared the pathogen, respectively

### GWAS for the death trait

**Fig. 2:**
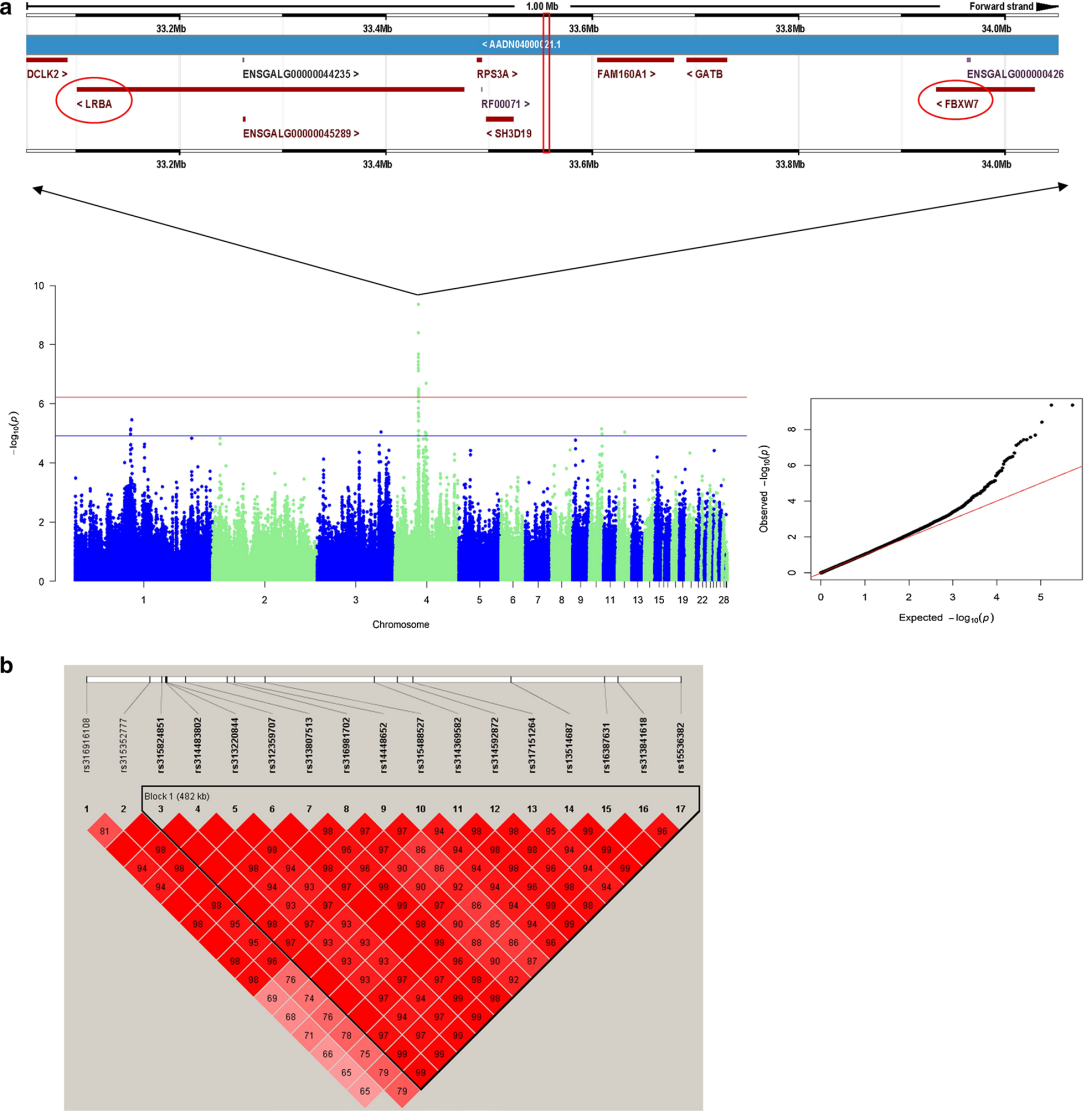
GWAS results for death. **a** Manhattan plot and Q–Q plot. Each dot on this figure corresponds to a SNP within the dataset, while the horizontal red and blue lines denote the genome-wide significance (6.88 × 10^−7^) and suggestive significance thresholds (1.38 × 10^−5^), respectively. The Manhattan plot contains − log10 observed P-values for genome-wide SNPs (y-axis) plotted against their corresponding position on each chromosome (x-axis), while the Q–Q plot contains expected − log10-transformed P-values plotted against observed − log10-transformed P-values. **b** LD plots for significant SNPs

The Manhattan and Q–Q plots for the death trait are in Fig. 2a. The single-marker analysis identified 42 SNPs that were significantly associated with death at the suggestive association threshold. Of these 42 SNPs, 17 genome-wide significant SNPs spanned a narrow 0.55 Mb region (33.48–34.03 Mb) on chicken chromosome GGA4 (GGA for *Gallus gallus*), which was the highest peak. Based on SNP annotation (Table 2), we found that 10 of the significant SNPs were located at intergenic regions, five were in introns and two were upstream of the coding sequences. Through a REML analysis implemented in the GCTA software, we found that the most significant SNP, i.e. rs314483802, (P = 4.38 × 10^−10^; intergenic) explained 11.73% of the phenotypic variance. An LD analysis revealed that all the genome-wide significant SNPs were in high LD (Fig. 2b), which makes it difficult to identify causal SNPs. The genomic control inflation factor (λ) calculated for the death trait was equal to 1.08, which is a little higher than the ideal value of 1 and indicates a mild but acceptable population stratification. We identified three genes that involved the eight upstream and intronic SNPs: *family with a sequence similarity 160 member A1* (*FAM160A1*), *F*-*box and WD repeat domain containing 7* (*FBXW7*), and *LPS responsive beige*-*like anchor protein* (*LRBA*). *FAM10A1* is a protein coding gene about which little is known in the literature. *FBXW7* modulates the NF-κB signaling pathway by targeting *NF*-*κB2* for ubiquitination and destruction [46, 47]. NF-κB is one of the most important signaling pathways of the inflammation and immune system. According to Fukushima et al. [47], the depletion of *Fbw7* (synonymous to *FBXW7*) in mice leads to reduced NF-κB activity and perturbed T cell differentiation. Thus, *FBXW7* is a very promising candidate gene that may affect immune response after SP infection. *LRBA* is an important gene that is involved in a syndrome of immune deficiency and autoimmunity. Deleterious mutations in *LRBA* cause defects in B cell activation and autophagy, and can increase susceptibility to apoptosis [48]. Furthermore, a new study has recently linked *LRBA* to the NF-κB immune pathway [49].

**Table 2:** Genome-wide significant SNPs for death and suggestive significant SNPs for carrier-state

Trait	SNP	GGA	Position	P-value	Annotation	Gene
Death	rs316916108	4	33,478,824	2.11E−08	Upstream	LRBA
	rs315352777	4	33,538,092	5.85E−07	intergenic	
	rs315824851	4	33,548,952	3.72E−08	Intergenic	
	rs314483802	4	33,552,203	4.38E−10	Intergenic	
	rs313220844	4	33,553,077	4.38E−10	Intergenic	
	rs312359707	4	33,554,042	3.72E−08	Intergenic	
	rs313807513	4	33,571,056	6.25E−08	Intergenic	
	rs316981702	4	33,610,050	3.96E−09	Intron	FAM160A1
	rs14448652	4	33,616,458	2.73E−08	Intron	FAM160A1
	rs315488527	4	33,644,936	4.80E−08	Intron	FAM160A1
	rs314369582	4	33,746,779	5.43E−07	Intergenic	
	rs314592872	4	33,768,246	3.61E−07	Intergenic	
	rs317151264	4	33,782,635	4.02E−07	Intergenic	
	rs13514687	4	33,873,598	7.70E−08	Intergenic	
	rs16387631	4	33,960,862	4.64E−07	Intron	FBXW7
	rs313841618	4	33,973,296	3.16E−07	Intron	FBXW7
	rs15536382	4	34,031,794	3.73E−07	Upstream	FBXW7
	rs313296110	4	43,491,648	2.53E−07	Intergenic	
	rs317612144	4	43,491,692	2.39E−07	Intergenic	
	rs318239967	4	43,491,742	3.30E−07	Intergenic	
	rs313303913	4	44,551,373	2.05E−07	Upstream	GPM6A
Carrier-state	rs312524326	5	50,147,152	9.52E−06	Intergenic	
	rs312970356	2	23,295,087	9.65E−06	Intergenic	
	rs317601331	5	49,848,247	1.09E−05	Intergenic	

**Fig. 3:**
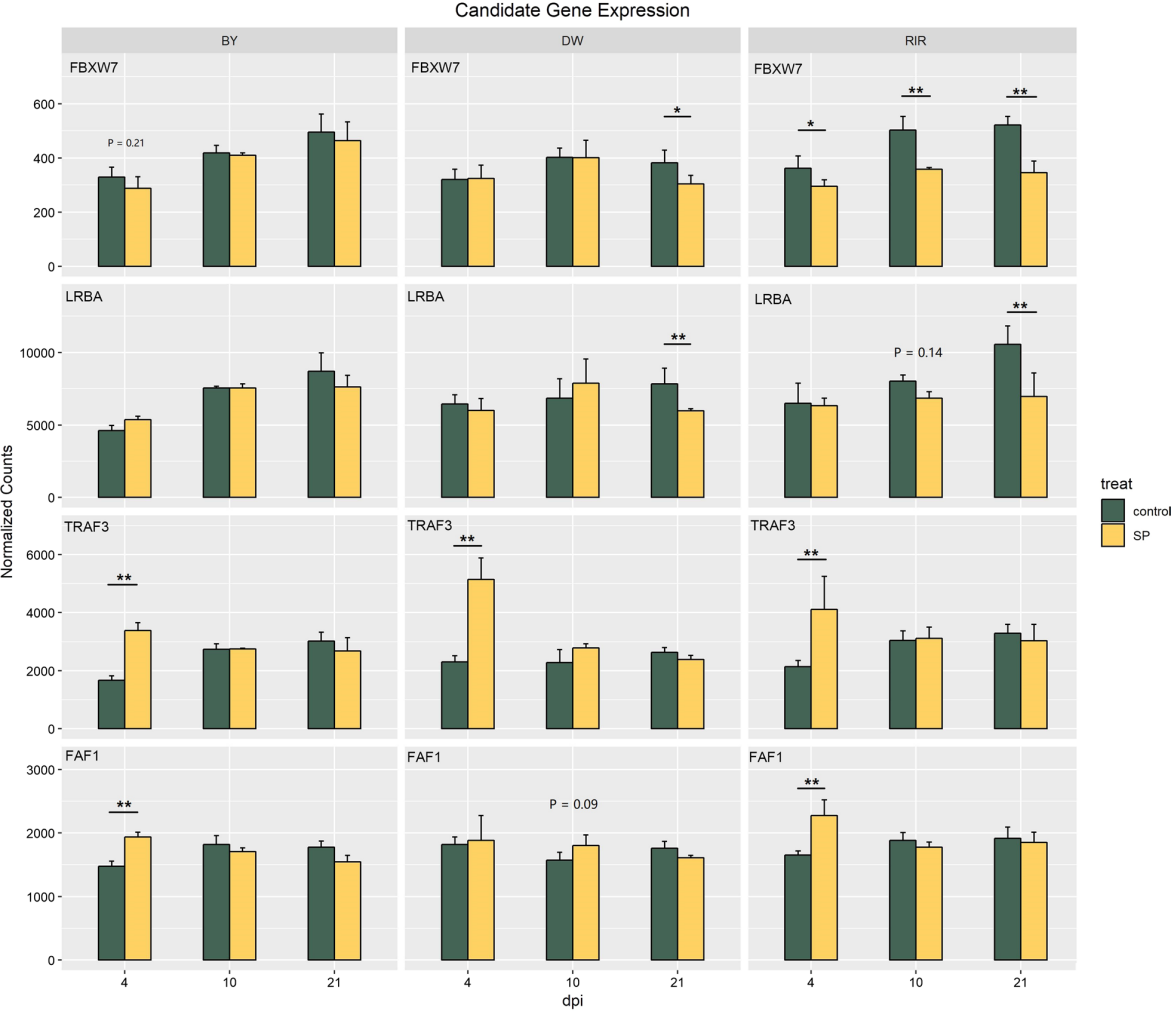
Candidate gene expression levels (normalized counts) in the three chicken lines (BY, DW and RIR) at three time points (4, 10, 21 dpi)

**Fig. 4:**
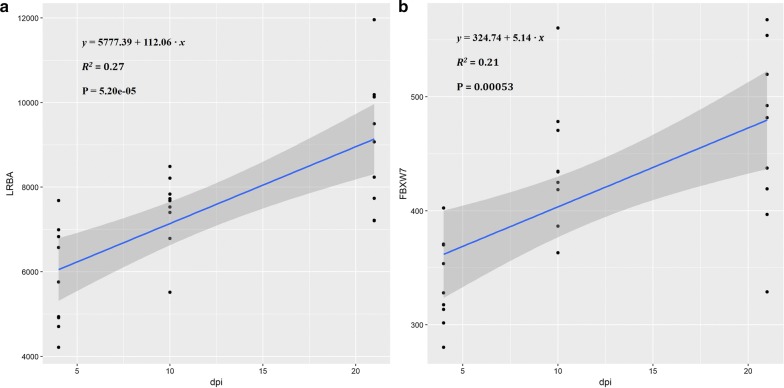
Regression analysis between gene normalized counts and time. The analysis was conducted using a linear model for **a** *LRBA*, *lipopolysaccharide*-*responsive and beige*-*like anchor protein* and **b** *FBXW7*, *F*-*box and WD repeat domain containing 7*

Our gene expression data provided further evidence that supports implication of these candidate genes during a SP infection. As shown in Fig. 3, expression of *FBXW7* was significantly downregulated after SP infection at all three time points in RIR chicks. Although this downregulation was not significant in BY chicks and was significant at 21 dpi in DW chicks, overall the same trend was observed in the three lines. Expression of *LRBA* was downregulated at a later time post-infection in all three lines. Interestingly, these two genes showed a time-dependent expression in the control group. To confirm this, we performed a regression analysis of gene expression levels and time (see Fig. 4) that showed that the expression levels of *LRBA* and *FBXW7* are positively correlated with time, which is consistent with the fact that the chicks are considerably more resistant to SP when they are more than 20 days old. Our results provide more evidence that *FBXW7* and *LRBA* are associated with resistance to SP.

### GWAS for the carrier state

**Fig. 5:**
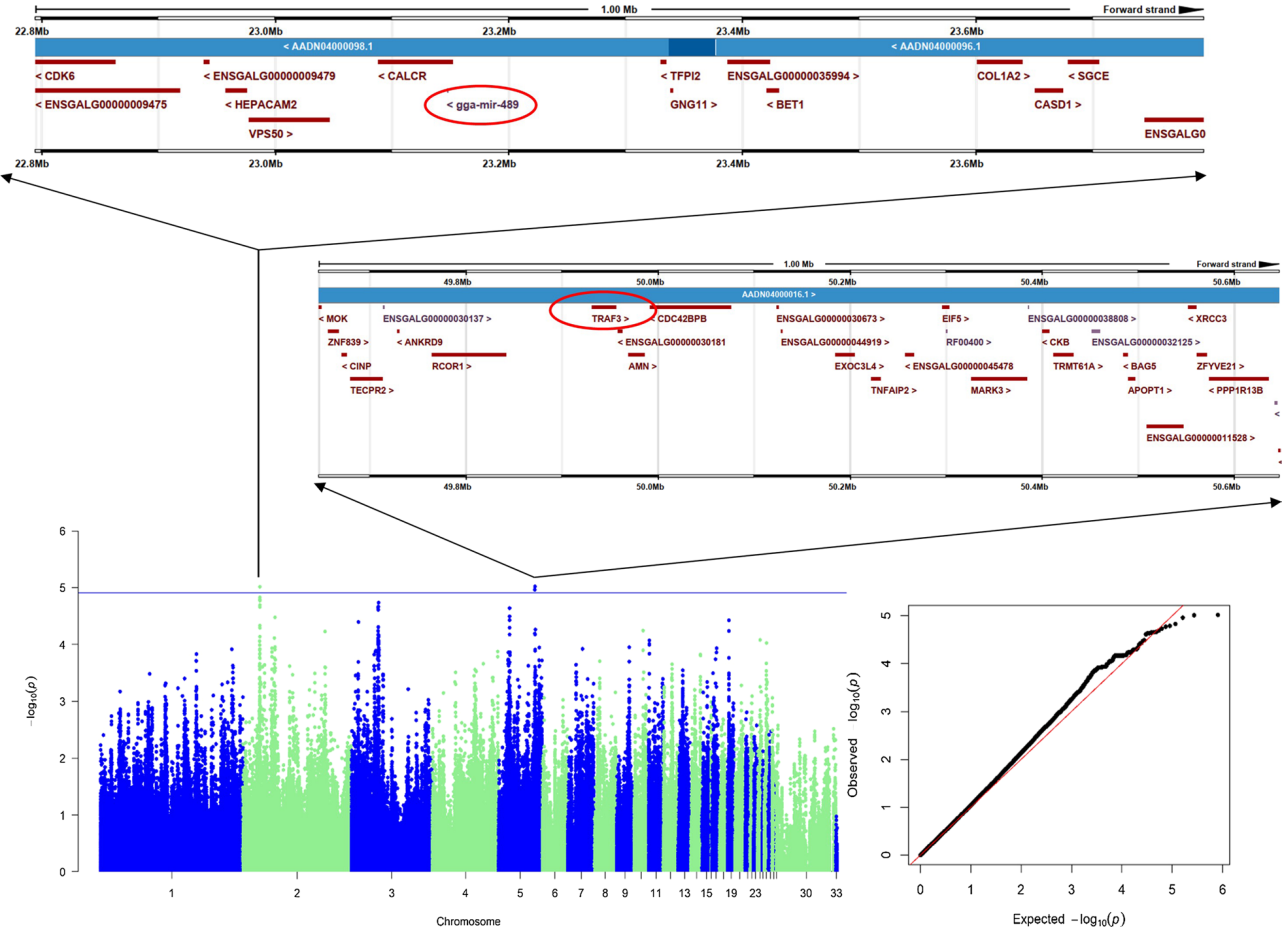
Manhattan plot and Q–Q plot for carrier-state. Each dot on this figure corresponds to a SNP within the dataset, while the horizontal blue line denotes the suggestive significance thresholds (1.38 × 10^−5^)

In the GWAS for the carrier-state trait, the linear mixed model GWAS could not identify genome-wide significant SNPs. However, three SNPs were above the level of suggestive significance (Fig. 5). The genomic control inflation factor (λ) for carrier-state was equal to 1.07, which is similar to the value found for death. Two of these three potential SNPs are located on GGA5 and the other one on GGA2. The rs312524326 SNP on GGA5 was located close to several candidate genes including the *TNF receptor associated factor 3* gene (*TRAF3*). *TRAF3* participates in the signal transduction of CD40, a member of the TNFR family that is important for the activation of immune response [50]. Besides, *TRAF3* controls the activation of the canonical and alternative NF-κB signaling pathway via the lymphotoxin beta receptor [51]. In our experiments, the expression level of *TRAF3* was up-regulated at 4 dpi in all three lines; however, it showed no difference at later times (Fig. 3). The SNP on GGA2 (rs312970356) was close to the miRNA *gga*-*mir*-*489*. Expression of *gga*-*mir*-*489* increases in CD30hi cells and is connected to Marek’s disease herpesvirus infection [52]. We used the miRDB tool [53, 54] to predict the target genes for *gga*-*mir*-*489* and found that one of the predicted target genes was *FAS associated factor 1* (*FAF1*) with a high target score of 98. The protein encoded by *FAF1* binds to the FAS antigen (TNFRSF6) and can initiate apoptosis or enhance apoptosis via the FAS antigen. Down-regulation of *FAF1* can activate the TNF-α/NF-κB signaling pathway [55]. We found that the expression of *FAF1* was up-regulated at 4 dpi in BY and RIR chicks after SP infection in our study (Fig. 3).

### Runs of homozygosity

**Fig. 6:**
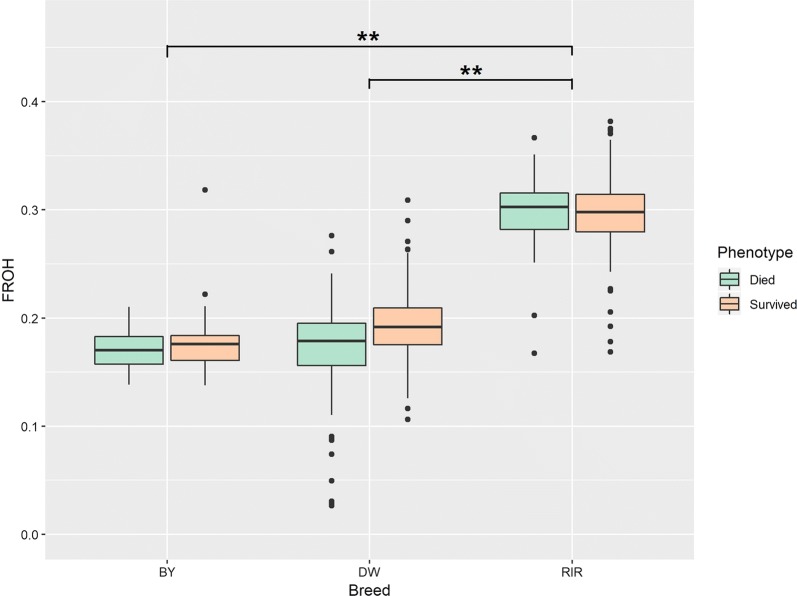
Genomic inbreeding coefficients (F_ROH_) in the three chicken breeds (** indicates P < 0.01)

We found that *F*_ROH_ did not differ between the traits analyzed here (Fig. 6), but the DW and BY chicks that died of SP infection showed the lowest values. In contrast, at the breed level, all comparisons of genomic inbreeding coefficient involving the RIR breed were significant.

## Discussion

*Salmonella* infections are a serious health risk to both humans and animals. In humans, typhoid fever outbreaks occur every few years and cause significant morbidity and mortality [56]. A human GWAS for *Salmonella* resistance conducted between infected individuals and a large control population reported a significant association with the MHC region [57]. Non-human research has the potential advantage of obtaining accurate phenotypes through bacterial challenge tests. However, only a few successful studies have been reported on infectious diseases owing to the difficulty in phenotyping the affected individuals and the complex genetic architecture of diseases. With the development of animal genomics, more domestic animals are being used as models for biomedical research [58].

To the best of our knowledge, this is one of the few large-scale GWAS, which have been carried out on both the death and carrier-state traits following a well-organized SP challenge experiment. We investigated the genetics of resistance to SP in 818 pure-bred chicks from three chicken lines by genotyping 302,927 SNPs from a high-density chip and performing a GWAS. Although difficult, it would be highly useful to find a general mechanism of disease resistance among populations; towards this aim, we used three chicken lines with different genetic backgrounds. We identified a strong association at a region that was located between 33.48 and 34.03 Mb on GGA4 for death, and two suggestive signals on GGA5 and GGA2 for carrier state. Combining the biological functions of the genes found in these regions and RNA-seq data, we identified four candidate genes that could account for resistance to SP, namely *FBXW7* and *LRBA* for death, and *TRAF3* and *gga*-*mir*-*489* (targeting *FAF1*) for carrier state. Interestingly, these four genes have been reported to participate in the NF-κB signaling pathway [47, 49, 51, 55], which suggests that this pathway may play a central role in the host’s resistance to SP infection. As a major transcription factor, NF-κB regulates many genes that are involved in both the innate and adaptive immune response [59]. Because SP is an intracellular bacterium, we suspect that the T cell development process related to the NF-κB signaling pathway may constitute the underlying mechanism of resistance to SP and thus deserves more research.

Until now, many *Salmonella* resistance genes have been identified in fowl [19], for example, *TLR4* and *SLC11A1*. However, most of these studies were carried out within a single population. A GWAS performed across multiple populations can increase the power to detect novel loci and achieve a higher mapping resolution than in previous studies [60–62]. The regions and gene candidates reported in our study have not been detected in previous studies on resistance to Salmonella infection, which may be explained by the fact that we used different chicken lines and *Salmonella* strains. The immunobiology of typhoidal and non-typhoidal Salmonella diseases differs substantially [63], and these diseases correspond to different host genetic resistances; thus, it is important to compare the mechanisms for different Salmonella diseases. Our study provided new data for the genetic determinism of typhoidal *Salmonella* resistance. Because disease resistance is a complex trait, it is likely that there are many more disease-related genes that could not be identified here due to the limited sample size.

The candidate genes identified in our study showed a time- and breed-related expression, which might be linked to different development stages of the affected individual. Analysis of the expression data reveals the complexity of the transcription of these genes at the different time points and in the different breeds. In the SP challenge test, the BY chicks were the most resistant to SP; however, RIR chicks (the most susceptible breed) showed more consistent results at different time points. Interestingly, the association between the candidate genes and resistance to SP was stronger in the RIR chicks with high susceptibility.

BY is a local breed of chickens with the highest level of resistance to the disease and the lowest inbreeding coefficient; DW is a synthetic line with both a low level of resistance to and a low inbreeding coefficient; RIR is the most intensively-selected line with a low level of resistance to SP and a high inbreeding coefficient. Many studies have reported that inbreeding is associated with disease resistance [43, 64–66], which is not the case in our study. One of the reasons could be that inbreeding depression in farm animals is more complex and different from that in wild populations.

Considerably more research is required before we can completely understand the genetics of any complex trait, especially traits that concern elaborate immunological functions. In this study, we show the feasibility of using a domestic animal for mapping the genomic regions that underlie an infectious disease and for providing new information that will be useful in subsequent comparative immunology studies. The genetic determinism of immunity is extremely complex and shaped by the contribution of multiple genes and environmental factors [67]. Therefore, more GWAS on domestic animals will help identify genes that are involved in the immunity mechanisms occurring during infectious diseases.

## Conclusions

Our GWAS on death and carrier state after SP infection identified new loci and genes associated with resistance to SP. The NF-κB signaling pathway is likely to play a central role in immunity against SP. These results provide new information on the genetic determinism of the resistance to infectious diseases; offer theoretical basis for breeding SP-resistant chicks using marker-assisted selection; and provide new data for research on salmonellosis in humans and other animals.

## Data Availability

The data on the 54 chickens used in RNA-Seq analysis are accessible at the National Center for Biotechnology Information (NCBI) under BioProject accession number PRJNA511038.

## Abbreviations

BY: Beijing You
DW: Dwarf Chicken
*FAF1*: FAS associated factor 1
*FBXW7*: F-box and WD repeat domain containing 7
GWAS: genome-wide association study
LD: linkage disequilibrium
LMM: linear mixed model
*LRBA*: lipopolysaccharide-responsive and beige-like anchor protein
NCBI: National Center for Biotechnology Information
NF-κB: nuclear factor kappa-light-chain-enhancer of activated B cells
PCA: principal component analysis
PCR: polymerase chain reaction
RIR: Rhode Island Red
RNA-Seq: RNA sequencing
SNP: single-nucleotide polymorphism
SP: *Salmonella pullorum*
*TRAF3*: TNF receptor associated factor 3
